# Challenges and opportunities for AMR research in the ASEAN following the One Health approach

**DOI:** 10.1016/j.onehlt.2025.101001

**Published:** 2025-02-27

**Authors:** Harish Kumar Tiwari, Daniel K.Y. Tan, Chhe Chinda, Duong Nu Tra My, Ha Thi Thu Hoang, Khao Keonam, Luu Quynh Huong, Ly Chanvatanak, Mot Virak, Nguyen Thuy Tram, Nittakone Soulinthone, Pham Duc Phuc, Thi Thu Hoai Nguyen, Vu Thi Thu Tra, Justin Beardsley

**Affiliations:** a Sydney Medical School, Faculty of Medicine and Health, University of Sydney, NSW, Australia; b School of Health Science and Technology, Indian Institute of Technology Guwahati, Guwahati, Assam, India; c DBT Wellcome Trust India Alliance Intermediate Fellow, Hyderabad, Telangana, India; d National Institute of Science Technology and Innovation, Cambodia; e Woolcock Institute, Viet Nam; f National Institute of Hygiene and Epidemiology, Viet Nam; g National University of Laos, Lao PDR, Laos; h National Institute of Veterinary Research, Viet Nam; i University of Puthisastra, Cambodia; j National Institute of Public Health, Cambodia; k Centre for Public Health and Ecosystem Research, Hanoi University of Public Health, Viet Nam; l International University, Vietnam National University of Ho Chi Minh City, Viet Nam; m Vietnam National University of Agriculture, Viet Nam

**Keywords:** Antimicrobial resistance, Southeast Asia, ASEAN, AMR. AMU, Challenges, Gaps

## Abstract

**Background:**

Antimicrobial resistance (AMR) has emerged as a significant global challenge and Southeast Asia with rapid economic and population growth faces substantial challenge in dealing with emerging infectious diseases and antimicrobial resistance. Here we present the recommendations of a workshop that explored the challenges and opportunities for One Health approach towards AMR research in three countries of AEAN, namely, Cambodia, Laos, and Vietnam.

**Methods:**

A workshop was organised in Hanoi, Vietnam in August 2023, involving participants involved in AMR research across varied sectors from three participating countries to prioritise the strategies that can be implemented in the region to fructify the One Health approach to tackle AMR. A modified Delphi approach was used to prioritise the top 10 Global Priority Research Questions for the region as developed by the Quadripartite (FAO, WHO, WOAH and UNEP). An iterative process was adopted to map priorities according to their impact and feasibility of application.

**Results:**

Collaborative initiatives, such as a common platform for listing the research goals, a web-based surveillance mechanism, and an enhanced AMR awareness curricula were identified as the steps forward. A consensus statement highlighting the critical needs for improved technical and infrastructure capacity, collaboration between sectors, increased funding, and systematic data analysis was drafted.

**Discussion:**

The participating countries have National Action Plans guided by the World Health Organization's Global Action Plan on AMR, but limited collaboration between human health and other sectors has impeded the benefits that One Health approach may achieve in the region. The recommendations include the need for improved technical and infrastructure capacity, and data collection across One Health sectors, besides increasing awareness at multiple levels.

**Conclusion:**

A collaborative and coordinated effort to apply One Health initiatives for tackling AMR in the ASEAN region is imperative. The workshop formulated a roadmap for future direction by identifying priorities aimed at enhancing collaboration, addressing infrastructure gaps, and contributing to an effective intervention in the fight against AMR in the region.

## 1 Introduction

The countries in the Southeast Asian region have witnessed rapid growth of their economies and population [[1], [2], [3]]. The development in the health systems has however not always kept pace and the region is recognised as a significant region for emerging infectious diseases and AMR [2,4]. Whilst improved availability and access to antimicrobials in the region has resulted in better therapeutic outcomes for infectious diseases, inappropriate usage has resulted in widespread AMR [[5], [6], [7], [8]]. Tackling of AMR through One Health approaches has shown positive results in many parts of the world but remains only partially applied in the ASEAN region [9,10]. There have been major investments in the workforce training and improving laboratory capacity, although these efforts have been isolated in space [[11], [12], [13]]. It is essential to have a collaborative and coordinated network of all agencies, institutes, individuals, and laboratories that have an integrated approach to tackling AMR in various countries across different disciplines.

Most countries of the region have formulated National Action Plans (NAP) supported by World health Organization Global Action Plan on AMR. There is a wealth of literature on the human drivers and spread of AMR in the region, informing solutions to counter the crisis such as improved stewardship, effective policy implementation, diagnostics, surveillance, and research [[13], [14], [15], [16], [17], [18], [19]]. Although Southeast Asian nations possess considerable infrastructure for surveillance and improving awareness of AMR/AMU along with capacity for diagnostics and stewardship, implementation is highly variable across countries and sectors. Integration with non-human health sectors and regional collaboration is still lacking, even though it is increasingly accepted that collaboration between nations and sectors can develop synergies, enhance capacity, and promote standardised research and diagnostic procedures [3,10,18].

The close relationships and shared bordered between Vietnam, Laos PDR, and Cambodia makes collaboration important. The food habits, climate and most agricultural practices are similar in the three countries. Moreover, the countries have close cultural relationships and are similar in socio-cultural and linguistic characteristics. Collectively, the region is a major supplier of primary food products, and hence there is a congruency in ensuring food security in the larger interest of export of food products ([20]; J. [21]; Jannie [22]). Further challenges in the Southeast Asia include over-supplied and unregistered drug outlets, a lack of qualified pharmacists, inadequate healthcare access, inappropriate antibiotic supply, and the need for multifaceted measures to align with the WHO's global action plan [8,23].

Despite the inherent strengths of similar geography and close socio-cultural ties to bolster AMR research in the region, the One Health approach is weakened by missing collaborations between the research institutes in the region and among the countries. As research in this domain being mostly donor-driven rarely aligning with the strategic needs of nations or the region, it is also affected by lack of expertise in human resources including the researchers, laboratory technicians, and clinicians [[24], [25], [26], [27], [28]]. Lack of coordination and capability gaps between the human, animal, and environmental sector in terms of infrastructure, finance, or/and technical expertise and indiscriminate use of antimicrobials without laboratory testing adds to the AMR in the region. There are fewer laboratories equipped with capacity to carryout routine AST (Antibiotic Sensitivity Test). Sharing of data among the laboratories and institutes engaged in similar research often results in duplicity of the efforts with small gain in scholarly output [29]. The research landscape in Vietnam, Cambodia, and Laos is rapidly evolving and complex. The emergence of AMR is already a significant public health threat and has increasing levels of political attention [10,14,30]. High levels of drug resistance have been identified in bacteria, fungi, parasites and viruses in Vietnam, Cambodia, and Laos [31], in ad-hoc surveillance studies, mostly conducted in human populations [32,33]. This paper identifies the challenges and opportunities for a potential One Health approach towards tackling the AMR situation in the Mekong Basin region of Southeast Asia.

From the 14–16 August 2023, we convened a workshop (funded by the University of Sydney Southeast Asia Centre (SSEAC) and supported by the Australian Department of Foreign Affairs and Trade (DFAT)), with the intention of exploring barriers and opportunities to implementing One Health AMR solutions in region, and developing a roadmap for progress. We discussed varying regulation and ways of operating in Cambodia, Laos PDR, and Vietnam to identify ways to leverage the One Health approach in tackling AMR in the region across various disciplines.

Discussions revealed, that despite the shared threat of AMR, the research landscape is characterised by limited collaboration, not only within the region but also between research sectors and institutes within countries. It was highlighted that research projects are mostly donor-driven and project based. Nonetheless, participants identified several factors promoting research potential. These include: similarities between countries in terms of AMR challenges, socio-cultural and behavioural characteristics, food habits, climate, and agricultural practices. The countries are all major suppliers of primary food products, so integrated control of AMR/AMU in the region makes sense and is a logical common goal. The need for an integrated approach to tackle AMR in the region has also been accentuated by the recent pandemic that has not only increased the uptake of antibiotics [8], but also resulted in outbreaks of previously controlled diseases due to failed or altered vaccination schedules and coverage [34].

Other problems highlighted by participants related to weaknesses in the One Health AMR research landscape. Despite high levels of political will, they recognised a relative lack of concrete examples of collaboration between sectors and institutes. They noted a lack of funding and especially funding flexible enough to support One Health AMR research. Furthermore, limits in technical expertise and research infrastructure mean that project start-up / capacity-building / training costs can be considerable. The lack of endogenous, sustainable funding means that training and capacity building costs are often not well-leveraged as staff move on to other projects or other types of work altogether. A considerable gap in capacity for research at the human, animal, and environmental interface was noted, both in terms of infrastructure, finance, and technical expertise.

Throughout the workshop, we focussed on the pillars and research priorities identified in the quadripartite report as a guide for assessing capacity and aspirations for One Health AMR research in the region.

## 2 Methodology

We conducted a series of webinars as part of a DFAT-funded project: ‘Networking One Health laboratories in ASEAN to tackle Antimicrobial Resistance’([23]). These webinars were attended by more than 70 researchers from the three countries and through round-table discussion and questionnaires that provided an opportunity for dialogue and collection of empiric data. Following the webinars, we sent a request for expressions of interest for a face-to-face workshop to all participants. The researchers who expressed their interest were forwarded the workshop agenda and the schedule for confirmation of their participation. The workshop was conducted in Vietnam 14–16 August 2023 and included participants from the three participating countries and 03 members from the SSEAC team, Australia (see Table 1).

**Table 1:** List of participants in the workshop held during 14–16 August 2023 to discuss ‘Challenges and opportunities for One Health approach to AMR research in Southeast Asia’.

Name	Country	Institution	Main area of research
Dr Ly Chanvatanak	Cambodia	University of Puthisastra,	AMR and AMS, Qualitative and quantitative study
Dr Chhe Chinda	Cambodia	National Institute of Science Technology and Innovation,	Food Science
Dr Mot Virak	Cambodia	National Institute of Public Health	Animal Health
Dr Pham Duc Phuc	Vietnam	Centre for Public Health and Ecosystem Research, Hanoi University of Public Health	Public health, One Health, infectious diseases, epidemiology, risk assessment
A/ Professor Thi Thu Hoai Nguyen	Vietnam	International University, Vietnam National University of Ho Chi Minh City	Public health, One Health, infectious diseases, epidemiology, risk assessment
Dr Vu Thi Thu Tra	Vietnam	Vietnam National University of Agriculture	Animal Health
Dr Duong Nu Tra My	Vietnam	Woolcock Institute	Public Health
Dr Nguyen Thuy Tram	Vietnam	National Institute of Hygiene and Epidemiology	Human Health
Dr Ha Thi Thu Hoang	Vietnam	National Institute of Hygiene and Epidemiology	Animal Health
Dr Luu Quynh Huong	Vietnam	National Institute of Veterinary Research	Animal Health
Dr Khao Keonam	Laos PDR	National University of Lao	Animal Health
Dr Nittakone Soulinthone	Laos PDR	National University of Laos	Veterinary medicine
Professor Daniel Tan	Australia	The University of Sydney	Agronomy, food safety, horticulture
Dr Harish Tiwari	Australia	The University of Sydney	Animal Health
Associate Professor Justin Beardsley	Australia	The University of Sydney	Fungal pathogens, One Health, community-based AMR interventions

The group thus assembled discussed the common themes drawn out of the literature review that were circulated as the discussion document. A thorough brain storming exercise led to identification of the key challenges, opportunities, and knowledge gaps facing AMR research following One Health approach in Vietnam, Cambodia, and Laos. A modified Delphi approach was used to contextualise and prioritise the Quadripartite's Top 10 Global Priority Research Questions to Vietnam, Cambodia, and Laos from the point of view of researchers with expertise in the field [[35], [36], [37], [38]]. The aim of this process was to identify the most important and feasible research needs in the region based on consensus among experts in the field. The modified Delphi approach ensured rigour and validity in the priority-setting process while maintaining inclusivity and transparency [[39], [40], [41], [42]]. The diverse group of experts in the workshop enabled capture of a range of perspectives from across disciplines, countries and academic sectors. The approach ensured that the final list of research questions was validated by experts in the field and that the prioritization process was driven by evidence and consensus.

The method involved a series of steps, starting with a pre-work stage in which all the experts involved in the study were provided with a copy of the Global Priority document. The experts were then asked to familiarize themselves with the research questions, the methods undertaken to develop them, and to consider the approaches that would be needed to answer them. In the first stage, visual analogues were used to rank the top 10 research questions from the global report based on their importance and feasibility/capability. The experts were asked to determine the importance and feasibility/capability of each question on a scale of 1 to 10, to determine where the questions place on a 2-dimensional matrix. The ranking of each question was then determined visually, through an iterative process. In the second stage, the experts participated in a structured discussion on the research priority areas. The experts revisited and re-ordered the ranking based on the overall priority of the research questions. The experts reached consensus through a process of discussion and debate to arrive at a final ranking of 10 research questions.

Throughout the workshop, we focussed on the pillars and research priorities identified the quadripartite report as a guide for assessing capacity and aspirations for One Health AMR research in the region.

## 3 Results and discussion

A total of 18 researchers including 03 from the University of Sydney participated in the workshop. The members from the University of Sydney participated in the brainstorming session and co-ordinated the modified Delphi sessions. The list of participants is presented in Table 1.

The top 10 research questions prioritized in the Quadripartite report were discussed extensively by the workshop participants and the final ranked list of priorities is presented in Table 2.

**Table 2:** Matrix representing ranking assigned consensually by the workshop participants about the research priorities in One Health approach to tackling AMR in Southeast Asia.

Ranking	Priority research question
1	How can One Health interventions that have proven impactful for AMR control and Mitigation most effectively be translated and scaled up in different context to differently resourced settings?
2	How can structural challenges and barriers to behaviours related to AMR be identified, characterised and assessed in different socio-cultural context?
3	What impacts the transmission of resistant microorganisms between humans, animals, plants and the environment, with a focus on conditions relevant to LMICs?
4	To what extent do various IPC practices in One Health settings impact the development and circulation of AMR in One Health sector?
5	What strategies can be used to adapt effective behavioural interventions (e.g. immunisation) from one context to another, (e.g. Africa to Asia/rural to urban/ human prescribers to veterinarians)?
6	What would a One Health AMR socio-economic impact assessment based on accurate and cost effectively collected data (e.g. harmonised methodology and indicators) in low resource settings optimally look like?
6	What challenges exist to the systematic collection and analysis of data for risk assessment and intervention impact assessment (epidemiological, economic, social) in LMICs?
7	How can governments identify, prioritise and institutionalise the most relevant crosscutting sector specific AMR policy options and regulatory frameworks and financing strategies to sustainably tackle AMR across One Health sectors, given their different implementation challenges?
8	How can existing AMR and AMU surveillance data from humans, Animals, plants and the environment be meaningfully triangulated and/or integrated to allow early identification of the development, escalation or circulation of resistance across One Health sectors?
9	What are the optimum strategies and minimum standards (and resources) for adequate laboratory and human resource capacity to establish and maintain quality integrated AMR surveillance systems at scale?

The common themes identified through the review of the literature prior to the workshop are listed below:

(a) Antimicrobial Resistance (AMR) is a significant public health concern with high levels of resistance found in Vietnam, Cambodia, and Lao PDR.
(b) Lack/ limited collaboration between research institutes in member countries, and hence, fewer collaborative publications.
(c) Although the policies and regulations on AMR (and AMU regulations) exist, implementation is limited at times.
(d) The existing research programs in the region are mostly donor-driven, and do not necessarily reflect strategic needs or strengths of the nations/region.
(e) AMR is not widely understood or prioritized at the policymaker level, and needs to be introduced in university and high-school curricula across disciplines such as health, agriculture, and environment.
(f) There is a need for more research into replacements for antibiotic use in both human and animal health sectors.
(g) There is a need to improve the laboratory capability to characterize organisms along with technical capacity building.

Discussions revealed, that despite the shared threat of AMR, the research landscape is characterised by limited collaboration, not only within the region but also between research sectors and institutes within countries. It was highlighted that research projects are mostly donor-driven and project based. Nonetheless, participants identified several factors promoting research potential. These include: similarities between countries in terms of AMR challenges, socio-cultural and behavioural characteristics, food habits, climate, and agricultural practices. The countries are all major suppliers of primary food products, so integrated control of AMR/AMU in the region makes sense and is a logical common goal. The need for an integrated approach to tackle AMR in the region has also been accentuated by the recent pandemic that has not only increased the uptake of antibiotics [8], but also resulted in outbreaks of previously controlled diseases due to failed or altered vaccination schedules and coverage [34]. Other problems highlighted by participants related to weaknesses in the One Health AMR research landscape. Despite high levels of political will, they recognised a relative lack of concrete examples of collaboration between sectors and institutes. They noted a lack of funding and especially funding flexible enough to support One Health AMR research. Furthermore, limits in technical expertise and research infrastructure mean that project start-up / capacity-building / training costs can be considerable. The lack of endogenous, sustainable funding means that training and capacity building costs are often not well-leveraged as staff move on to other projects or other types of work altogether. A considerable gap in capacity for research at the human, animal, and environmental interface was noted, both in terms of infrastructure, finance, and technical expertise.

Based on these extensive discussions, several additional challenges were highlighted by participants. The countries of Southeast Asia present both challenges and opportunities for academics and scientists engaged in research on One Health AMR research. The foremost challenge in this domain is the is the lack of data on social science, behavioural, and economic-policy factors, which form the foundational basis for conducting research in this area [3,9,43]. Additionally, data collection proves to be an arduous task as accessing technical expertise and diagnostic equipment, especially for antimicrobial susceptibility testing is not organised and in some instances completely lacking [44,45]. The existing standards for laboratory methods, protocols, and availability of robust data for analysis is not consistent, hence comparison among countries is difficult [46,47].

Further, laboratory issues related to quality control and quality assurance can make it difficult to obtain accurate results and can also be a challenge when it comes to affordable access to important testing equipment [44,48,49].Translating research into behavioural changes and measuring the impact of policy changes due to variable enforcement can be challenging [44,48,49]. Another challenge is the lack of a national or regional database with consistent data on AMR across One Health sectors, which can hamper the ability to monitor the issue or conduct research. It can also be challenging to access rural and remote regions, which can limit research participation among vulnerable communities [19,24]. Besides, implementation of the interventions also pose a formidable challenge as boosting knowledge of AMR in policymakers, communities, and educational curricula involves coordination among multiple stakeholders at local and national level [7].

Notwithstanding the numerous challenges to addressing the Quadripartite research questions that AMR researchers in the region encounter, some opportunities were also identified. These include: increasing funding for AMR globally, strengthening government support for One Health AMR research through National Action Plans (NAPs), and including gender, indigenous, and vulnerable populations in research, which can improve translatability of proposed interventions [14,18]. The participants felt that in addition to the identified challenges there are many unanswered questions, or unexplored fields for investigation that have significant potential impact and can be good areas for future research. Infection Prevention Control (IPC) interventions have not yet been fully explored and can also be an area for research in the region. In addition, there are significant amounts of data that can be linked for immediate impact in the region. Overall, it is important for researchers to consider the challenges and opportunities when conducting One Health AMR research in the region with an aim to address the existing challenges and capitalize on the opportunities to inform effective interventions and policies resulting in reduced burden of infections, and antibiotic resistance.

## 4 A vision for future AMR research in Southeast Asia

Our vision for One Health AMR research in Vietnam, Cambodia, and Laos includes several key elements that promote collaboration, joint funding, networking, knowledge and capacity sharing, capacity building, and sustainability.

One of the key elements of the vision involves ensuring that research funds are sufficient and sufficiently flexible to support the research work needed. Joint funding initiatives across the region are vital to ensuring this vision can become reality. Joint funding will promote collaboration among researchers, policymakers, and other relevant stakeholders to address the challenges related to AMR. Another important aspect of the vision is to ensure action plans on AMR for the region are integrated and based on evidence and best practices. This will ensure that policy is informed by data and research is conducted in a coordinated manner across different sectors and countries according to need.

Another element of the vision is to promote connectivity between laboratories to share data and research with a common database specifically for AMU/AMR. This will enable researchers to collaborate more effectively and efficiently, and it will also promote knowledge and capacity sharing across different sectors and countries. Ideally this will extend beyond the three countries in the region.

The vision also includes conducting research on alternatives to antimicrobials (e.g., vaccines) especially in human health and animal husbandry practices. This will contribute to the goal of promoting a more sustainable use of antimicrobials and reducing resistance to antibiotics. Additionally, capacity building for biosecurity and biosafety will ensure that research activities are conducted in a safe and secure manner. Finally, the vision includes promoting collaboration between sectors - public and private - and sustained funding support from governments, to ensure that the research work is sustainable over the long term.

Overall, the vision for One Health AMR research in Vietnam, Cambodia, and Laos is a holistic approach that promotes collaboration, joint funding, networking, knowledge and capacity sharing, capacity building, and sustainability.

## 5 Key takeaways from the workshop and steps

The key takeaways from the workshop are:

(a) Improving technical and infrastructure capacity to conduct One Health AMR research is needed across the region.
(b) Collecting more data on AMR across one health sectors is important, but understanding and improving systems was prioritized over this.
(c) One Health AMR work is gaining academic and political interest, but much of the infrastructure is not permissive, and engagement of broad stakeholders is important to increase the profile of AMR.
(d) Strengthening One Health policies in NAPs, increasing research output and profile, and collaborative networks are also important next steps.

The workshop highlighted concrete steps to advance research in the region. The participants suggested the following steps to promote research on One Health approach towards tackling AMR in the region:

Some highlighted concrete steps to advance research in the region, included:

(a) A common platform for sharing research goals.
(b) Conducting research on transmission of AMR across sectors as a prime priority.
(c) The need to explore ways to help governments prioritise and regulate One Health AMR research through policies.
(d) A common web-based surveillance mechanism to rate AMR in the region.
(e) Bridging the gap in expertise and lab infrastructure and capacity across all sectors.
(f) Sharing researchers, students, and joint research projects.
(g) Establishing mechanisms for data sharing, including material transfer agreements (MTAs)
(h) Enhanced AMR awareness curriculum at various levels, including schools and universities
(i) Systematic analysis and meta-analysis of available research data for joint publication

Promote further workshops and conferences to network, exchange information, and further understanding among scientists in the region.

## 6 Conclusion

The rise of antimicrobial resistance (AMR) has been a consistent threat to global public health, especially considering the COVID-19 crisis with reduced vaccines in human and animal health, leading to increased hospitalizations and outbreaks of diseases such as rabies in Vietnam and Laos, and diphtheria in Laos. This workshop has highlighted the urgent need for education and engagement on Antimicrobial Resistance and One Health at both the community, school, university, and continuing professional development (CPD) level. Additionally, the private/commercial sector must also be engaged. The region is technologically advanced, and there is great promise in innovative technological solutions for Antimicrobial Stewardship (AMS) across sectors.

A comprehensive understanding of the drivers of Antimicrobial Use (AMU) is necessary for developing strategies to reduce its misuse and abuse. Collaborative research networks between sectors and countries are required to address this issue, and the development of standard operating procedures, best practices, and the implementation of global goals are crucial for improving the accuracy and effectiveness of the research results.

Finally, there is a pressing need for governments to develop strategies for working together across sectors, such as the ministries of health, agriculture and fisheries, industry, and others, to share best practices and promote One Health in the region. Not only must we produce more data, but we also need to ensure that the data are appropriate, cohesive, and aligned with global goals.

In conclusion, this workshop has served as a platform for dialogue and collaboration between various stakeholders in the One Health domain, highlighting the need for urgent action to reduce Antimicrobial Resistance and strengthen global One Health networks. We call on the international community, including governments, research organizations, and industry leaders, to prioritise and fund collaborative research networks and education and engagement efforts that will help us address this global crisis and improve public health outcomes for all.

The workshop proceedings highlighted several initiatives that can strengthen the One Health network in the ASEAN region, nonetheless, it was limited by smaller participation and lack of representation of stakeholders from the agriculture sector and also the private players such as pharmaceutical companies. The scope of the proceedings is restricted by monetary compulsions and hence could not involve greater participation. That the workshop participants were drawn from an earlier project to establish network of laboratories working in AMR research in Cambodia, Laos PDR, and Vietnam [23], some crucial aspects of regional cooperation such as economic impact of AMR in the region could not be discussed. Despite these limitations, the recommendations of the workshop can be instrumental in laying foundation for initiating opportunities for mutual cooperation in AMR research not limited to participating countries but for the entire ASEAN region.

## 7 Consensus statement

One Health AMR research is a highly critical and cross-sectoral area that requires collaborative partnerships and a shared understanding of the challenges and opportunities for progress. The following consensus statement highlights the key challenges and opportunities identified by stakeholders in the workshop and sets out a roadmap for One Health AMR research moving forward.

• We acknowledge the critical need for improved technical and infrastructure capacity to conduct One Health AMR research. This includes access to technical expertise and diagnostic equipment, standardization of laboratory methods and data analysis platforms for comparison across sectors and countries, and increased engagement of rural and remote communities in research.
• We encourage collaboration between sectors for both research and implementation and stress the importance of building partnerships to address AMR and One Health.
• We recognize the need for increased funding for One Health AMR research and strong government support through NAPs, and the importance of including gender, indigenous and vulnerable populations in research.
• We recognize the value of existing data and the importance of developing a common platform for sharing research goals to enhance collaboration.
• We acknowledge the need to address the challenges of restricted access to technical expertise and equipment, and to promote One Health and AMR knowledge among policymakers and communities.
• We endeavour to use mutual networks to engage researchers from other domains.
• We agree that systematic and meta-analysis of available research data and their joint publication are important steps in advancing research in the region.

This consensus statement provides a roadmap for addressing the challenges and opportunities for One Health AMR research in the region. The priorities outlined here will help guide our efforts towards achieving better patient and public health outcomes.

## Funding source

Sydney Southeast Asia Centre Workshop Grant to Harish Kumar Tiwari. The funding source had no involvement in the study design, data collection, interpretation, writing of the manuscript, or decision to submit the article for publication.

## CRediT authorship contribution statement

**Harish Kumar Tiwari:** Writing – review & editing, Writing – original draft, Resources, Methodology, Funding acquisition, Data curation, Conceptualization. **Daniel K.Y. Tan:** Writing – review & editing, Supervision, Investigation. **Chhe Chinda:** Writing – review & editing, Investigation. **Duong Nu Tra My:** Writing – review & editing, Resources, Investigation. **Ha Thi Thu Hoang:** Writing – review & editing, Resources, Investigation. **Khao Keonam:** Writing – review & editing, Investigation. **Luu Quynh Huong:** Writing – review & editing, Resources, Investigation. **Ly Chanvatanak:** Writing – review & editing, Investigation. **Mot Virak:** Writing – review & editing, Investigation. **Nguyen Thuy Tram:** Writing – review & editing, Investigation. **Nittakone Soulinthone:** Writing – review & editing, Investigation. **Pham Duc Phuc:** Writing – review & editing, Investigation. **Thi Thu Hoai Nguyen:** Writing – review & editing, Investigation. **Vu Thi Thu Tra:** Writing – review & editing, Resources, Investigation. **Justin Beardsley:** Writing – review & editing, Writing – original draft, Supervision, Methodology, Funding acquisition, Formal analysis, Conceptualization.

## Declaration of competing interest

The authors declare that there are no conflicts of interest regarding the publication of this article.

## Data Availability

Data will be made available on request.
